# Fucoidan Derived from *Undaria pinnatifida* Induces Apoptosis in Human Hepatocellular Carcinoma SMMC-7721 Cells via the ROS-Mediated Mitochondrial Pathway

**DOI:** 10.3390/md11061961

**Published:** 2013-06-10

**Authors:** Lili Yang, Peisheng Wang, Huaxin Wang, Qiaomei Li, Hongming Teng, Zhichao Liu, Wenbo Yang, Lin Hou, Xiangyang Zou

**Affiliations:** 1 Department of Biotechnology, Dalian Medical University, Dalian 116044, China; E-Mails: yll_0504@126.com (L.Y.); WPS4585@126.com (P.W.); whx@dlmedu.edu.cn (H.W.); qiaomei@126.com (Q.L.); thm0529@126.com (H.T.); caredliu@sina.com (Z.L.); saliva@163.com (W.Y.); 2 College of Life Science, Liaoning Normal University, Dalian 116081, China

**Keywords:** apoptosis, fucoidan, hepatocellular carcinoma, reactive oxygen species

## Abstract

Fucoidans, fucose-enriched sulfated polysaccharides isolated from brown algae and marine invertebrates, have been shown to exert anticancer activity in several types of human cancer, including leukemia and breast cancer and in lung adenocarcinoma cells. In the present study, the anticancer activity of the fucoidan extracted from the brown seaweed *Undaria pinnatifida* was investigated in human hepatocellular carcinoma SMMC-7721 cells, and the underlying mechanisms of action were investigated. SMMC-7721 cells exposed to fucoidan displayed growth inhibition and several typical features of apoptotic cells, such as chromatin condensation and marginalization, a decrease in the number of mitochondria, and in mitochondrial swelling and vacuolation. Fucoidan-induced cell death was associated with depletion of reduced glutathione (GSH), accumulation of high intracellular levels of reactive oxygen species (ROS), and accompanied by damage to the mitochondrial ultrastructure, depolarization of the mitochondrial membrane potential (MMP, Δ*ψ*m) and caspase activation. Moreover, fucoidan led to altered expression of factors related to apoptosis, including downregulating *Livin* and *XIAP* mRNA, which are members of the inhibitor of apoptotic protein (IAP) family, and increased the Bax-to-Bcl-2 ratio. These findings suggest that fucoidan isolated from *U. pinnatifida* induced apoptosis in SMMC-7721 cells via the ROS-mediated mitochondrial pathway.

## 1. Introduction

Fucoidans are a class of fucose-enriched sulfated polysaccharides primarily extracted from brown seaweeds [1,2]. It has recently been reported that fucoidans possess a wide variety of biological activities, including anticoagulant, antiviral, anti-angiogenic, anticancer and immunomodulatory activities [3,4]. In particular, the anticancer activity of fucoidans has attracted considerable attention. Several investigations have demonstrated that fucoidans can effectively suppress proliferation and colony formation by cancer cells *in vitro* [5], and inhibit metastasis and angiogenesis of Lewis lung adenocarcinoma and B16 melanoma xenografts *in vivo* [6,7]. Compared to other sulfated polysaccharides, the fucoidan extracted from the sporophylls of the brown seaweed *Undaria pinnatifida* has a higher sulfate and l-fucose content, and exhibits a broader range of bioactivities [8].

Hepatocellular carcinoma (HCC) is the third leading cause of cancer-related deaths, which has high morbidity and mortality rates [9,10]. Due to the accumulation of several genetic and epigenetic changes within the tumor cells, HCC has a relatively low therapeutic selectivity and high drug resistance, and these major issues reduce the efficacy of chemotherapy in patients with this disease [11].

Apoptosis, or programmed cell death, is an important aspect of chemotherapy-induced tumor cell death; and is the major mechanism of tumor cell death induced by many anticancer drugs and natural products [12]. Caspase-dependent apoptosis is characterized by activation of either the extrinsic pathway, initiated by activation of death receptors leading to the cleavage of caspase-8, or the intrinsic pathway, triggered by mitochondrial depolarization, release of cytochrome c and the subsequent activation of caspase-9 [13,14]. Disruptions to the factors regulating these apoptotic pathways contributes substantially to the transformation of a normal cell into a tumor cell, and the cells of some tumor types are relatively resistant to apoptosis [15,16].

Intracellular reactive oxygen species (ROS) are considered to be an apoptotic death signal [17]. However, low physiological levels of ROS also serve as a signaling messenger to mediate various biological responses, including cell proliferation, angiogenesis, innate immunity, gene expression, apoptosis and senescence [18]. It has also been established that increased levels of these short-lived reactive molecules can exert harmful effects by inducing oxidative damage to biological macromolecules and disrupting the cellular reduction-oxidation (redox) balance. Such disturbances to ROS homeostasis are generally considered to be a risk factor for the initiation and progression of diseases such as atherosclerosis, neurodegeneration and cancer [19]. ROS induce depolarization of the mitochondrial membrane potential (MMP, Δ*ψ*m) and the release of cytochrome c from the mitochondria into the cytosol, where cytochrome c triggers activation of caspase-9 and initiates the caspase cascade, which ultimately induces the cell to undergo apoptosis [20]. Tumor cells are more sensitive to fluctuations in the levels of ROS than normal cells; therefore, ROS are also considered as an important target in anticancer drug research [21,22].

The present study was designed to evaluate the anticancer effects of the fucoidan extracted from *U. pinnatifida* sporophylls in human HCC SMMC-7721 cells, and investigate the molecular mechanisms of these effects.

## 2. Results and Discussion

### 2.1. Preparation and Properties of *U. pinnatifida* Fucoidan

The fucoidan extracted and purified from *U. pinnatifida* sporophylls was a beige, fibrous powder (purity > 90%). Infrared spectrum and ^13^C-NMR analyses of the sample revealed strong characteristic absorption peaks for sulfated residues, fucose and galactose, respectively. The sample mainly consisted of carbohydrates (68.37%), sulfates (21%) and uronic acid (10.89%), with fucose and galactose mainly constituting the monosaccharide component; the percentage protein content was determined to be 0.85%. The molecular weight of the purified fucoidan was approximately 10.4356 × 10^4^ Da. The optical rotation of the fucoidan (0.6 mg/mL) at 20 °C was 0.99°.

### 2.2. Fucoidan Induces Apoptosis in SMMC-7721 Cells

**Figure 1:**
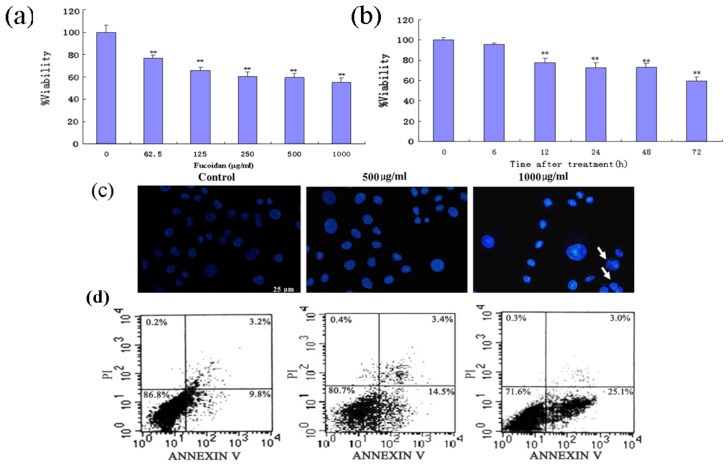
Effects of fucoidan on cell viability and apoptosis in SMMC-7721 hepatocellular carcinoma (HCC) cells. (**a** and **b**) Viability of cells treated with various concentrations of fucoidan (65.2–1000 μg/mL) for 48 h (**a**) or with 500 μg/mL fucoidan for 6, 12, 24, 48 or 72 h (**b**) as determined by the MTT assay. Data is the mean ± SD of at least three independent experiments; * *P* < 0.05, ** *P* < 0.01 compared with control cells. (**c**) Fucoidan induces apoptosis in SMMC-7721 cells. Nuclear morphology, as indicated by Hoechst 33258 staining. (**d**) Quantification of apoptosis by the annexin V/PI double staining assay using flow cytometry. LL (low left), LR (low right), UR (upper right), and UL (upper left) denote viable (live), early apoptotic, late apoptotic and necrotic cells, respectively.

To investigate the effects of the fucoidan in human HCC cells, SMMC-7721 cells were exposed to various concentrations of the fucoidan for up to 72 h, and then subjected to 3,(4,5-dimethylthiazol-2-yl)-2,5-diphenyltetrazolium bromide (MTT) assay. The fucoidan inhibited SMMC-7721 cell viability in a concentration- and time-dependent manner (Figure 1a,b). Fucoidan-induced SMMC-7721 cell death was confirmed by Hoechst 33258 staining, and annexin V/propidium iodide (PI) staining by flow cytometry. Nuclear fragmentation and chromatin condensation, the typical morphological characteristics of apoptotic cells, were observed in fucoidan-treated cells stained with Hoechst 33258 (Figure 1c); however, these features were rarely observed in control cells. Annexin V/PI double-staining and flow cytometry revealed that fucoidan effectively induced apoptosis in SMMC-7721 cells (Figure 1d). The proportion of apoptotic cells (lower right quadrant) significantly increased from 9.8% in untreated cells to 14.5%–25.1% in fucoidan-treated cells. The proportion of necrotic cells did not significantly change after exposure to fucoidan.

### 2.3. Effect of Fucoidan on Cell Cycle Distribution in SMMC-7721 Cells

**Figure 2:**
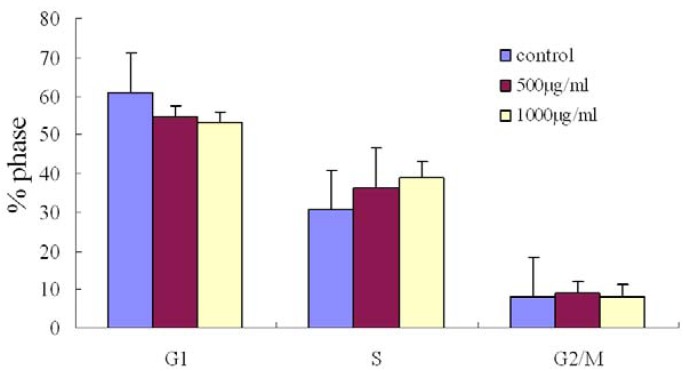
Changes in the cell cycle distribution in SMMC-7721 cells treated with fucoidan. The cell cycle trended towards accumulation of S phase cells in the cells treated with fucoidan (500 μg/mL or 1000 μg/mL for 24 h).

To determine the effects of the fucoidan on the cell cycle, the cells were treated with the fucoidan for 48 h, then the percentage of cells in each phase of cell cycle was determined by using a flow cytometry. SMMC-7721 cells treated with fucoidan (1000 μg/mL) for 48 h tended to accumulate in the S-phase; however, this increase was not significant compared to control cells (Figure 2).

### 2.4. Fucoidan-Induced Apoptosis in SMMC-7721 Cells Is Dependent on Caspase Activation

**Figure 3:**
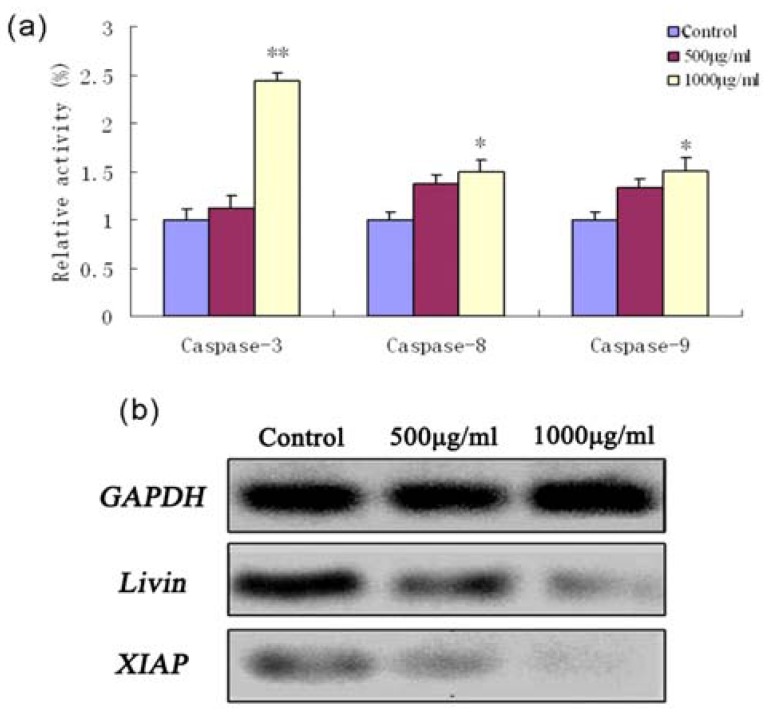
Caspase activation and expression of inhibitor of apoptotic protein (IAP) proteins during fucoidan-induced apoptosis in SMMC-7721 cells. (**a**) Activation of caspase-3, caspase-8 and caspase-9 were examined using commercial colorimetric assays and expressed as relative activities. The relative activities of caspase-3, caspase-8 and caspase-9 in SMMC-7721 cells treated with fucoidan for 24 h were higher than that of untreated control cells. (**b**) RT-PCR analysis of *Livin* and *XIAP* mRNA expression. *GAPDH* was examined as an endogenous control.

To assess whether the fucoidan-induced apoptosis in SMMC-7721 cells was caspase-dependent, the enzyme activities of initiator (-8 and -9) and effector (-3) caspases were analyzed using commercial colorimetric assays. As shown in Figure 3a, 1000 μg/mL fucoidan induced activation of caspase-3 (*P* < 0.01), caspase-9 and caspase-8 (*P* < 0.05). Moreover, fucoidan lead to downregulation of *XIAP* and *Livin* mRNA expression; these proteins are endogenous inhibitors of caspase-3 and caspase-9 (Figure 3b).

### 2.5. Effect of Fucoidan on Mitochondrial Morphology in SMMC-7721 Cells

**Figure 4:**
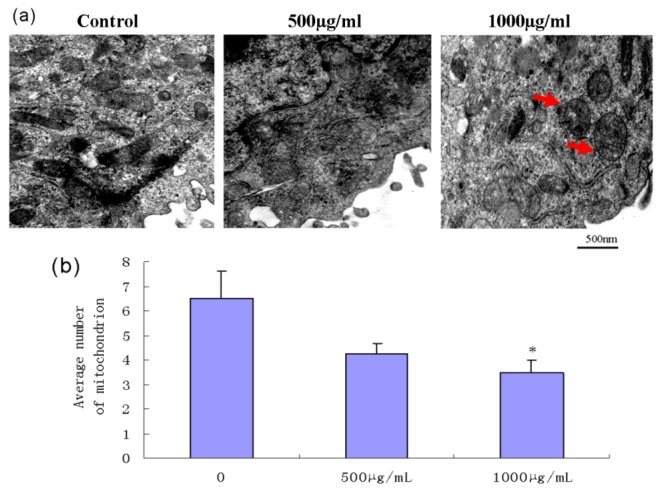
Ultrastructural changes in mitochondrial morphology in SMMC-7721 cells after exposure to fucoidan. (**a**) After 24 h treatment with fucoidan, SMMC-7721 cells exhibited mitochondria swelling and vacuolation, as observed by transmission electron microscopy. Arrows indicate swollen mitochondria. (**b**) The average number of mitochondria was counted in a 2000 nm × 2000 nm square. Mitochondrial number was significantly lower in the cells treated with fucoidan (1000 μg/mL) than control cells (* *P* < 0.05).

The changes in mitochondrial ultrastructure were observed by transmission electron microscopy. Cells treated with fucoidan for 24 h exhibited the typical features of apoptotic cells, including swelling and vacuolation of the mitochondria (Figure 4a). The average number of mitochondria was determined in a square area of 2000 nm × 2000 nm. The average mitochondrial number in cells treated with fucoidan was significantly less than that in control cells (Figure 4b).

### 2.6. Fucoidan Induces Mitochondrial Dysfunction and Increases the Bax/Bcl-2 Ratio in SMMC-7721 Cells

**Figure 5:**
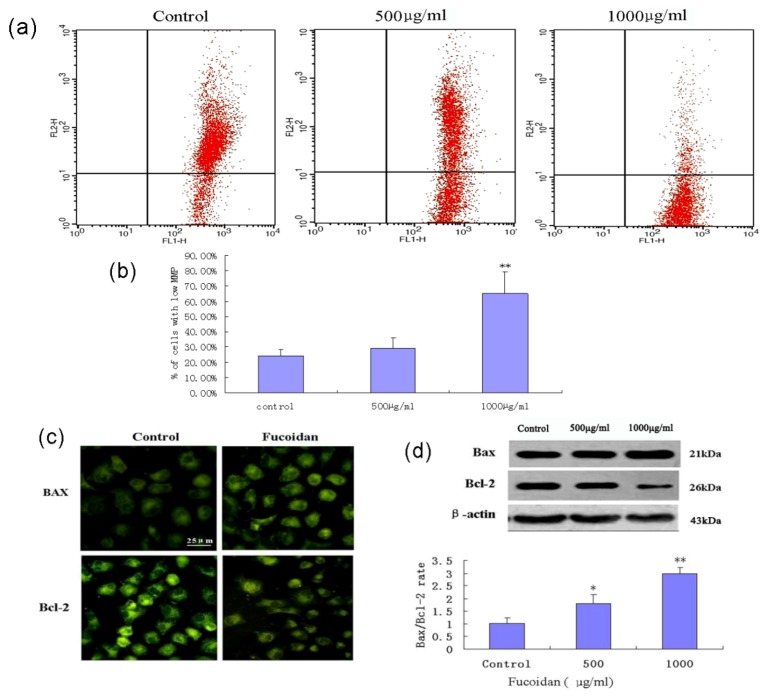
Fucoidan induces mitochondrial dysfunction and increases the Bax/Bcl-2 ratio in SMMC-7721 cells. (**a**) Depolarization of mitochondrial membrane potential (MMP, Δ*ψ*m) was examined in control SMMC-7721 cells and SMMC-7721 cells exposed to fucoidan (500 μg/mL or 1000 μg/mL for 24 h); the cells were stained with JC-1 and analyzed by flow cytometry. (**b**) Percentage of cells with a low MMP (Δ*ψ*m). Data are mean ± SD of three independent experiments; ** *P* < 0.01 compared with control cells. (**c**) Immunocytochemical localization of Bcl-2 and Bax. SMMC-7721 cells were treated with fucoidan, fixed, permeabilized with 0.2% Triton X-100 and subjected to immunofluorescent staining using anti-Bcl-2 and anti-Bax antibodies. Microphotographs were taken using a fluorescence microscope. (**d**) Western blotting analysis of Bcl-2 and Bax expression, and the Bax/Bcl-2 ratio in the cells exposed to fucoidan (500 μg/mL or 1000 μg/mL for 24 h) or unexposed. * *P* < 0.05, ** *P* < 0.01 compared to controls. Data from optical density measurements were tested using one-way ANOVA.

Mitochondrial function was evaluated by measuring mitochondrial membrane potential (MMP, Δ*ψ*m) using the fluorochrome JC-1 and flow cytometry. Fucoidan treatment of SMMC-7721 cells induced significant dissipation of the MMP (Figure 5a) in a concentration-dependent manner (Figure 5b). Immunocytochemistry and western blotting analysis revealed that fucoidan downregulated expression of the anti-apoptotic protein Bcl-2, and moderately increased expression of the pro-apoptotic protein Bax. Fucoidan induced a concentration-dependent increase in the Bax/Bcl-2 ratio in SMMC-7721 cells (Figure 5c,d).

### 2.7. Fucoidan Induces Mitochondrial Release of Cytochrome c in SMMC-7721 Cells

**Figure 6:**
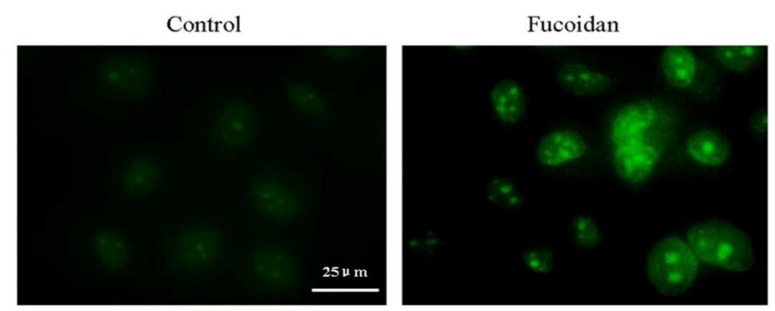
Fucoidan induces the release of cytochrome c from the mitochondria in SMMC-7721 cells. Immunocytochemical localization of cytochrome c. SMMC-7721 cells were treated with fucoidan (1000 μg/mL) for 24 h, fixed, permeabilized with 0.2% Triton X-100 and subjected to immunofluorescent staining using a cytochrome c antibody. Microphotographs were taken using a fluorescence microscope.

Release of cytochrome c from the mitochondria to cytosol was detected using an immunofluorescent method. The results showed that treatment of SMMC-7721 cells with fucoidan induced the release of cytochrome c from the mitochondria into the cytosol (Figure 6).

### 2.8. Fucoidan Induces an Intracellular ROS Burst and GSH Depletion in SMMC-7721 Cells

**Figure 7:**
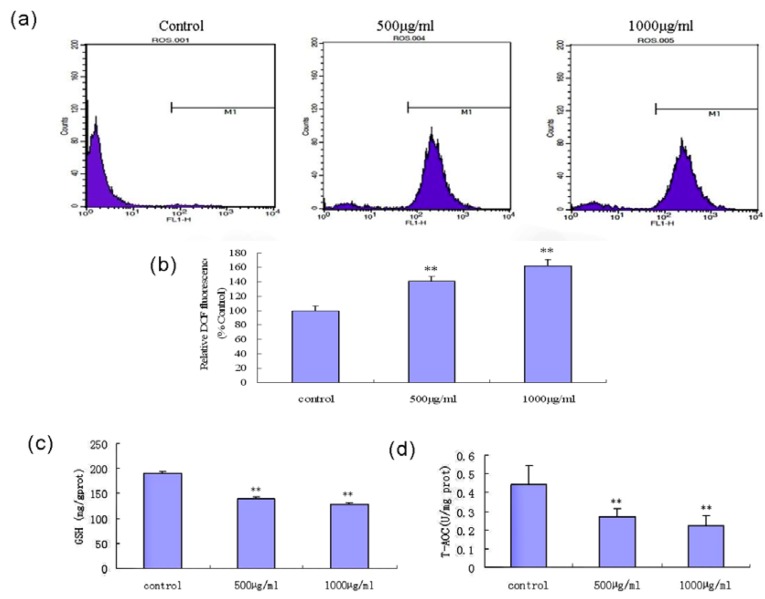
Fucoidan induces intracellular reactive oxygen species (ROS) generation, and reduces glutathione GSH) content and total anti-oxidant capability (T-AOC) in the cells. (**a**) Levels of ROS in SMMC-7721 cells following exposure to fucoidan. Cells were treated with 500 μg/mL or 1000 μg/mL fucoidan for 24 h, then stained with detection of the fluorescent probe (DCFH-DA) commercial colorimetric kit for flow cytometric analysis. (**b**) Relative fluorescent intensity of DCFH-DA in fucoidan-treated cells, expressed as a percentage of the control group. (**c**, **d**) Generation of intracellular GSH and T-AOC were determined in cells treated with fucoidan for 24 h. ** *P* < 0.01 compared to control cells.

The generation of intracellular ROS in fucoidan-treated SMMC-7721 cells was monitored by detection of the fluorescent probe (DCFH-DA) by flow cytometry. As shown in Figure 7a,b, exposure to fucoidan triggered the production of ROS in a concentration-dependent manner. To determine whether the changes in ROS accumulation were related to GSH depletion or a decline in total anti-oxidant capability (T-AOC), we investigated the effects of fucoidan on intracellular GSH and T-AOC in SMMC-7721 cells using commercial colorimetric assays. Fucoidan partially depleted the intracellular GSH content (Figure 7c) and led to a decrease in cellular T-AOC in the cells (Figure 7d).

### 2.9. Discussion

Fucoidans are potent inducers of apoptosis in various cancer cell lines. The fucoidan derived from the sporophylls of *U. pinnatifida* has a higher sulfate and l-fucose content than the fucoidans extracted from other brown seaweeds. *U. pinnatifida* fucoidan has previously been shown to possess anti-cancer, anti-proliferative and anti-coagulative activities [8]. Several studies have also demonstrated that fucoidans from several brown algae can activate the extrinsic or intrinsic apoptotic pathways in a variety of cancer cell lines by altering the expression of apoptosis-associated or signaling proteins, cell cycle regulatory proteins and transcription factors [23,24,25,26]. Although these fucoidan has been shown to induce apoptosis in SMMC-7721 HCC cells, the molecular and cellular mechanism(s) underlying these effects had not yet been determined.

In the present study, exposure of SMMC-7721 cells to the fucoidan isolated from *U. pinnatifida* sporophylls resulted in apoptotic cell death, accompanied by nuclear fragmentation, vacuolization of the mitochondria, depolarization of the MMP, release of cytochrome c from the mitochondria and caspase activation. Furthermore, the apoptosis induced by the fucoidan in SMMC-7721 cells was related to the generation of ROS, depletion of intracellular GSH, a decrease in T-AOC, an increase in the Bax/Bcl-2 ratio, and downregulation of *XIAP* and *Livin*. These observations suggest that the fucoidan induces apoptosis in SMMC-7721 cells via the ROS-mediated mitochondrial pathway.

The ability to induce cellular apoptosis is an important property of many candidate anticancer drugs [27]. Apoptosis is a tightly regulated process, which involves at least one of the caspase-dependent signaling pathways: the cell death receptor pathway or the mitochondrial pathway [28]. In the mitochondrial pathway, a variety of death signals trigger the release and translocation of several pro-apoptotic proteins from mitochondria to cytosol. Among the numerous factors known to modulate apoptosis in cancer cells, the proteins of the Bcl-2 family are viewed as the main regulators of apoptosis, and investigation of their function has been the focus of intensive research for more than twenty years [29]. Bcl-2 is an anti-apoptotic protein, whereas Bax is a crucial pro-apoptotic and tumor suppressor protein [30,31]. Bcl-2 also plays an important role in the regulation of mitochondrial energetics, transport of adenine nucleotides, Ca^2+^ and other metabolites, and mitochondrial membrane permeability [32]. The ratio of anti-apoptotic to pro-apoptotic molecules, such as the Bcl-2/Bax ratio, indicates the threshold sensitivity of cells to the induction of apoptosis via the intrinsic pathway [33]. In our experiment, the protein expression levels of Bcl-2 and Bax were assessed. Our results indicated that the fucoidan downregulated Bcl-2 protein expression, upregulated Bax protein expression, and increased the Bax/Bcl-2 ratio in a concentration-dependent manner in SMMC-7721 cells.

Activation of caspases is a pivotal step in the apoptotic process, and is triggered by signals from death factors, mitochondrial alterations or DNA damage due to external and/or internal insults [34]. Regardless of whether apoptosis occurs by the cell death receptor pathway or mitochondrial pathway, both pathways ultimately activate caspase-3, which in turn induces DNA fragmentation, the characteristic morphological change associated with apoptotic cells; activation of caspase-3 indicates a key and irreversible point in the induction of apoptosis [35]. Depolarization of MMP induced by apoptotic stimuli often leads to mitochondrial permeability transition pore formation, which enables the release of cytochrome c from the mitochondrial inter-membrane space into the cytosol [36], which in turn triggers the activation of caspase-9 and induces apoptosis via the caspase-dependent mitochondrial pathway [37]. In the present study, exposure to the fucoidan resulted in depolarization of the MMP, release of cytochrome c from the mitochondria, and activation of caspase-3, caspase-8 and caspase-9 in SMMC-7721 cells. Of the members of the IAP protein family, XIAP has been reported to exert the strongest anti-apoptotic function, as it inhibits caspase-3, caspase-7 and caspase-9 [38]. Livin also inhibits the activation of caspase-9 induced by cytochrome c, Apaf-1 and dATP [39]. This study demonstrated that the fucoidan downregulated the expression of *XIAP* and *Livin* mRNA, which was associated with activation of caspase-3 and caspase-9, indicating that fucoidan-induced apoptosis occurred via the mitochondrial pathway. Our results also suggest that the fucoidan-induced apoptosis also involved co-activation of the caspase-8 and caspase-9-mediated pathway.

Oxidative stress refers to an imbalance between pro-oxidant and anti-oxidant factors, which are controlled by multiple components; such imbalances may lead to cellular damage. ROS play a key role in oxidative stress, and are generated as by-products of cellular metabolism, primarily in the mitochondria [40]. Once accumulated, ROS can attack cellular proteins, DNA and lipids, which leads to a state of oxidative stress. ROS contribute to a number of human diseases including cardiovascular, inflammatory and neurodegenerative diseases, as well as cancer. Elevated levels of mitochondrial ROS have been shown to be sufficient to trigger apoptosis [41]. In this study, the apoptotic effect of the fucoidan in SMMC-7721 cells was associated with a rapid increase in the levels of intracellular ROS; after treatment with the fucoidan for 24 h, the levels of ROS significantly increased. Additionally, the fucoidan-induced ROS generation was associated with a significant depletion of intracellular GSH, which is a major non-protein cellular antioxidant which can eliminate intracellular ROS [42]. The degree of exposure to ROS and perturbations to the GSH redox balance play a critical role in determining whether cells undergo a pro-survival or pro-death response [43]. A 10% reduction in the GSH content can induce apoptosis in a variety of cancer cells, including lung cancer, hepatoma and breast cancer cells, but has almost no effect in normal cells. Tumor cells have a significantly higher sensitivity to changes in the levels of GSH, due to the fact that tumor cells have a heightened basal level of ROS-mediated signals which contributes to their increased rate of growth, metabolism and proliferation; therefore, tumor cells may be more vulnerable to oxidative stress [22]. In this study, the total cellular antioxidant capacity (T-AOC) was remarkably decreased in fucoidan-treated SMMC-7721 cells. Fucoidan might exhausted the total cellular antioxidant capacity and increased the ROS levels beyond a “threshold”, which may have contributed to the induction of apoptosis in SMMC-7721 cells. Similar findings with regards to cell cycle arrest were reported to be induced by the fucoidan from Mozuku seaweed (*Cladosiphon novae*-*caledoniae* Kylin) in MCF-7 breast cancer cells [44].

Taken together, our findings demonstrate that the fucoidan induces apoptosis in SMMC-7721 cells via the ROS-mediated mitochondrial pathway, by increasing ROS production, inducing mitochondrial oxidative damage, MMP depolarization and release of cytochrome c, combined with downregulation of *XIAP* and *Livin* and activation of caspase-3 and caspase-9. We suggest that the potent pro-apoptotic effects of the fucoidan be due to its high content of sulfate groups, which resulted in depletion of intracellular GSH, which in turn triggered mitochondrial oxidative damage and the activation of caspase-9 and caspase-3. This study suggests that marine fucoidans may represent candidate anti-cancer drugs. Further research is required to investigate how fucoidans enter tumor cells, and to determine whether they are absorbed directly by the tumor cells and/or accumulate in tumor tissues.

## 3. Experimental Section

### 3.1. Reagents and Antibodies

RPMI 1640 medium, antibiotics (penicillin and streptomycin), trypsin-EDTA, dimethylsulfoxide (DMSO) and fetal bovine serum (FBS) were obtained from Hyclone (Logan, UT, USA). MTT reagent and trypsin were purchased from Sigma (St. Louis, MO, USA). Mouse anti-Bcl-2, Bax and cytochrome c IgG monoclonal antibodies and rabbit anti-β-actin IgG polyclonal antibody were purchased from Santa Cruz Biotechnology (Santa Cruz, CA, USA). Horseradish peroxidase-conjugated anti-rabbit and anti-mouse IgG were purchased from Beijing Zhongshan Biotechnology Co., Ltd. (Bejing, China). FITC-conjugated rabbit anti-mouse IgG was obtained from Thermo Fisher Scientific (NY, USA). The human cytochrome c ELISA kit was obtained from Bioleaf Biotechnology Co., Ltd. (Shanghai, China). Annexin V-FITC, propidium iodide (PI) and Hoechst 33258 were supplied by KeyGen Biological Technology Co., Ltd. (Nanjing, China).

### 3.2. Preparation and Analysis of Fucoidan

The fucoidan was prepared from sporophylls of *U. pinnatifida* by trypsin-enzymatic hydrolysis and alcohol grade precipitation. Infrared spectra and ^13^C-NMR spectra were recorded on Nicolet 510P spectrophotometer (Nicolet Co., USA) and Brucker AV500 NMR instrument (Brucker Co., Sweden), respectively. The total carbohydrate, sulfate radical and uronic acid contents were measured by the phenol-sulfuric acid reaction, BaCl_2_-gelation and sulfuric acid-carbazole colorimetric methods, respectively. The molecular weight of the sample was evaluated by size exclusion chromatography using TSK-gel G 3000 PWXL (TOSOH, Tokyo, Japan). Optical rotation was measured at 589 nm using the WZZ-1 polarimeter (Yemao Co., Ltd., Shanghai, China) at 20 °C. Above tests were performed as previously reported [4].

### 3.3. Cell Culture

The human HCC cell line SMMC-7721 was obtained from the Cell Bank of the Chinese Academy of Sciences (Beijing, China). SMMC-7721 cells were cultured in RPMI 1640 medium containing 10% FBS, 100 μg/mL penicillin and 100 μg/mL streptomycin. The cells were incubated at 37 °C in a humidified incubator (Thermo Fisher Scientific, New York, NY, USA) in an atmosphere of 5% CO_2_.

### 3.4. Cell Viability Assay

The effects of fucoidan on SMMC-7721 cell viability were measured using the MTT assay. Logarithmically-growing SMMC-7721 cells were seeded at a density of 2 × 10^4^ cells/well in 96-well plates and allowed to adhere for 24 h at 37 °C. Then, the supernatant was replaced with 200 μL culture medium supplemented with different concentrations of fucoidan. The cells were incubated for 6, 12, 24, 48 or 72 h, then 20 μL MTT (5 mg/mL) was added to each well, incubated for an additional 4 h at 37 °C, the medium was removed, the formazan crystals were dissolved in 150 μL DMSO, and the absorbance values were measured at wavelength of 490 nm using a microplate reader (Thermo Fisher Scientific).

### 3.5. Cell Cycle Analysis

The cell cycle distribution of cells treated with fucoidan for 24 h or 48 h was assayed by measuring the DNA content of nuclei labeled with propidium iodide (PI). Briefly, the cells were harvested, fixed in 70% ethanol for 24 h, incubated with 50 μg/mL PI and 0.25 mg/mL RNase A at 37 °C for 30 min, and then analyzed using a FACSCalibur™ Flow Cytometer (BD Biosciences, San Jose, CA, USA) with excitation at 488 nm and detection at 620 nm. Data was gated to exclude cellular debris. The proportions of G1, S and G2-M phase cells were calculated from the DNA content histograms.

### 3.6. Apoptosis Assay

SMMC-7721 cells were seeded into 6-well plates (3 × 10^5^ cells /well), allowed to adhere for 24 h, and treated with 500 μg/mL or 1000 μg/mL fucoidan for 24 h. The cells were collected, washed twice with chilled PBS and stained using annexin V-FITC labeling solution (annexin V-fluorescein in binding buffer containing PI (KeyGen)) according to the manufacturer’s protocol. Apoptotic cells were analyzed by using a FACSCalibur flow cytometry.

### 3.7. Hoechst 33258 Staining

The cells treated with fucoidan were stained with Hoechst 33258 (4 μg/mL) for 30 min, fixed for 10 min in 4% *para*-formaldehyde (PFA), and then observed by DMI-4000B inverted fluorescence microscopy (Leica, Germany).

### 3.8. Transmission Electron Microscopy

SMMC-7721 cells were treated with fucoidan for 24 h, washed with PBS, centrifuged (1000 rpm for 10 min), and the cell pellets were fixed in 2.5% glutaraldehyde in pH 7.2 sodium cacodylate buffer overnight. The cells were post-fixed in 1% osmium tetroxide, dehydrated through an ascending alcohol series, embedded in Epon resin and sectioned using an ultramicrotome. Ultrathin sections were stained with saturated uranyl acetate and aqueous lead citrate, and examined by transmission electron microscopy using a JEM-1220 (JEOL, Japan) operating at 60 kV.

### 3.9. Assay of Mitochondrial Membrane Potential

Mitochondrial membrane potential (Δ*ψ*m) was measured using the mitochondrial membrane sensor kit containing the dye JC-1 (Nanjing KeyGEN Biotech. Co., Ltd., Nanjing, China), as described by the manufacturer. The cells were treated with or without fucoidan for 24 h, and then harvested for flow cytometric analysis.

### 3.10. Immunofluorescence Staining

The cells were suspended in RPMI 1640 medium and transferred to 6-well plates (3 × 10^5^ cells/well) culture dished with sterile cover slips and grown up to 60% confluency, and the cells were treated with or without fucoidan for 24 h. Cells grown on cover slips washed with cold PBS, fixed for 15 min with 4% PFA, permeabilized with 0.2% Triton X-100 in PBS for 5 min, blocked with 0.1% BSA, and then incubated with the appropriate primary antibody in 1% BSA at room temperature, detected by the appropriate fluorescence-conjugated anti-rabbit or anti-mouse IgG antibody for 15 min. After washing with PBS, the cover slips were mounted of coverslides on glass slides and analyzed under using a LSM510 fluorescence microscope (Carl Zeiss, Germany).

### 3.11. Caspase Activation Assays

Caspase-3, caspase-8 and caspase-9 activation was determined using Caspase Colorimetric Assay kits (KeyGen) following the manufacturer’s instructions. The background absorbance value was subtracted from the absorbance results of the test wells. These experiments were performed independently three times.

### 3.12. Measurement of Intracellular ROS, GSH Levels and T-AOC

ROS generation was monitored by staining cells with DCFH-DA using T-AOC detection assay kit (KeyGen). Briefly, following exposure to fucoidan, the cells were incubated with 5 μM DCFH-DA at 37 °C for 30 min, trypsinized, washed with PBS and the fluorescence intensity of the cells was analyzed immediately by flow cytometry using FI-1 filters at an excitation wavelength of 488 nm. Generation of intracellular reduced glutathione (GSH), an index of cellular reducing power, and total antioxidant capability (T-AOC) were measured using the kits following the manufacturer’s instructions.

### 3.13. Western Blot Analysis

Cells were collected, washed twice with cold PBS, lysed using lysis buffer (KeyGen) for 20 min on ice, and centrifuged at 14,000 *g* for 20 min at 4 °C. The supernatant of the protein lysates was subjected to SDS-PAGE using 12% gel, electro-transferred onto nitrocellulose membranes, incubated with the appropriate specific primary and secondary antibodies, and the bands were detected using chemiluminescence.

### 3.14. Semi-Quantitative RT-PCR Analysis

For RT-PCR analysis of *Livin* and *XIAP* mRNA expression, total RNA was isolated from the cells using TRIzol (Invitrogen, Carlsbad, CA, USA) and cDNA was synthesized according to the manufacturer’s instructions (TaKaRa, Japan). The sequences of the forward and reverse primers were: 5′-CGTCTTGGTTCTTCCCA-3′ and 5′-GTTCCCCAGCTGTCAGTTC-3′ for *Livin*; 5′-TGTCCCTTCTGTTCTAACAG-3′ and 5′-GCAGGGTTTCTTTATACTGG for *XIAP*; and 5′-CGCGAGAAGATGACCCAGAT-3′ and 5′-GCACTGTGTTGGCGTACAGG-3′ for *GAPDH*. PCR analysis was performed under the following conditions: denaturation at 94 °C for 5 min, followed by 35 cycles of denaturation for 30 s at 94 °C, annealing for 30 s at 55 °C and extension for 60 s at 72 °C. The amplified products were analyzed by 1% agarose gel electrophoresis followed by ethidium bromide staining. *GAPDH* served as a loading control.

### 3.15. Statistical Analysis

Statistical analysis of the data was performed using SPSS 11.5 software. Each experiment was carried out twice with triplicate measurements for quantitative comparisons, and data are expressed as the mean ± SD values. The Student’s *t*-test was used to determine the significance of the differences in multiple comparisons; * *P* < 0.05 was considered statistically significant.

## 4. Conclusions

Taken together, our findings demonstrate that fucoidan induces apoptosis in SMMC-7721 cells via the ROS-mediated mitochondrial pathway, by increasing ROS production, inducing mitochondrial oxidative damage, MMP depolarization and release of cytochrome c, combined with downregulation of *XIAP* and *Livin* and activation of caspase-3 and caspase-9. *U. pinnatifida* fucoidan might have potential as a candidate marine drug for human hepatocarcinoma.
